# Associations of maternal serum concentration of iron-related indicators with birth outcomes in Chinese: a pilot prospective cohort study

**DOI:** 10.1186/s13052-024-01621-0

**Published:** 2024-03-05

**Authors:** Geng-dong Chen, Peng-sheng Li, Zi-xing Zhou, Hai-yan Wang, Xiao-yan Gou, Shao-xin Ye, Dong-xin Lin, Da-zhi Fan, Li-juan Wang, Zheng-ping Liu

**Affiliations:** 1 Foshan Institute of Fetal Medicine, Foshan Women and Children Hospital, 528000 Foshan city, Guangdong Province China; 2 Department of Obstetrics, Foshan Women and Children Hospital, No.11 Renmin West Road, Changchen District, 528000 Foshan City, Guangdong Province China; 3 Biobank of Foshan Institute of Fetal Medicine, Foshan Women and Children Hospital, 528000 Foshan, Guangdong China

**Keywords:** iron-related indicators, Femur length, Birth weight, Pregnancy, Birth outcome

## Abstract

**Background:**

Previous studies of maternal iron and birth outcomes have been limited to single indicators that do not reflect the comprehensive relationship with birth outcomes. We aimed to investigate the relationship between maternal iron metabolism and neonatal anthropometric indicators using comprehensive iron-related indicators.

**Methods:**

A total of 914 Chinese mother-child dyads were enrolled in this prospective study. Subjects’ blood samples were collected at ≤ 14 weeks of gestation. Serum concentrations of iron-related indicators were measured by enzyme-linked immunosorbent assay (ELISA). Femur length was measured by B-ultrasound nearest delivery. Neonatal anthropometric indicators were collected from medical records.

**Results:**

After adjustment for potential covariates, higher iron (per one standard deviation, SD increase) was detrimentally associated with − 0.22 mm lower femur length, whereas higher transferrin (per one SD increase) was associated with 0.20 mm higher femur length. Compared with normal subjects (10th-90th percentiles), subjects with extremely high (> 90th percentile) iron concentration were detrimentally associated with lower femur length, birth weight, and chest circumference, and a higher risk of low birth weight, LBW (HR: 3.92, 95%CI: 1.28, 12.0). Subjects with high concentration of soluble transferrin receptor, sTFR and transferrin (> 90th percentile) were associated with higher femur length. Subjects with low concentration of iron and ferritin concentrations (< 10th percentile) were associated with a higher risk of LBW (HR: 4.10, 95%CI: 1.17, 14.3) and macrosomia (HR: 2.79, 95%CI: 1.06, 7.35), respectively.

**Conclusions:**

Maternal iron overload in early pregnancy may be detrimentally associated with neonatal anthropometric indicators and adverse birth outcomes.

**Supplementary Information:**

The online version contains supplementary material available at 10.1186/s13052-024-01621-0.

## Background

With the assistance of pediatric clinicians and researchers, infant mortality has significantly decreased in the last century. However, challenges persist in other critical areas of infant health, such as fetal growth restriction (FGR) [1, 2]. Pediatricians have long known that iron deficiency (ID) contributes to anemia long before [1]. Recent research indicates that ID may have a negative impact on cognitive development during early childhood [3]. Additionally, it has been linked to socioemotional development in both infancy and adolescence [4]. Maternal nutrition during pregnancy can have an impact on fetal growth and development. Maternal nutrition during pregnancy has been linked to child development. For instance, supplementing with folic acid during pregnancy reduces the risk of neural tube defects [5]. Maternal malnutrition of critical nutrients can affect fetal programming, leading to an increased risk of FGR and long-term consequences of chronic disease in adulthood [2]. The effects of iron deficiency anemia on mothers and infants have received significant attention. Iron deficiency anemia or iron deficiency has long been known to have detrimental effects on fetal growth and development, including fetal anthropometric indicators such as birthweight, head circumference, and body length [6–8]. Iron deficiency anemia has been a persistent global concern that requires resolution [9]. In recent years, however, some studies have suggested that excessive iron intake, or iron overload, may have adverse health effects, such as an increased risk of Parkinsonism [10] and type 2 diabetes [11]. During pregnancy, iron overload may also have adverse effects. For example, excessive iron supplementation may increase the risk of gestational diabetes [12]. However, research on the effects of iron overload during pregnancy on fetal growth and development is still limited.

Several studies have investigated the relationship between maternal iron status (including dietary intake, iron supplementation, and serum concentration of iron indicators) and fetal or neonatal growth. However, the results of these studies have been inconsistent and heterogeneous. In a meta-analysis of 48 randomized controlled trials (RCTs) and 44 cohorts, it was found that prenatal iron supplementation was associated with higher birth weight and a lower risk of low birth weight (LBW) compared to no supplementation [6]. Several studies have found positive associations between iron supplementation [13], dietary iron intake [14–16], and iron concentration in blood [17], cord blood [18], and placenta [8], and neonatal anthropometric indicators such as body length [8, 19, 20], chest circumference [21], and head circumference [7, 18]. Two systematic reviews [22, 23] did not find any significant associations between iron supplementation (mostly combined with folic acid) and anthropometric indicators in infants, children, and adolescents. These results were further supported by several other studies investigating iron exposure from dietary intake [24], blood [25], and amniotic fluid [26]. Conversely, several studies have suggested potential adverse associations between higher blood or placental iron status and anthropometric indicators [27–31]. Two studies of South Korean women found that higher serum ferritin concentration or dietary iron intake were associated with lower femur length and biparietal diameter [27, 28]. Similar negative associations were observed in children between higher iron status (or higher hemoglobin levels) and head circumference [29], birth weight [30, 32, 33], and height [31].

Several flaws in the previous studies require careful consideration. The investigation of iron exposure exhibited significant heterogeneity, encompassing dietary intake (foods or supplements), intervention with iron supplements (single or combined with folic acid or multiple micronutrients), and serum iron status (single or multiple indicators). Bio-indicators provide more accurate data than dietary surveys and are not influenced by factors such as individual dietary absorption rates. Additionally, several laboratory biochemical parameters are associated with anemia [3]. However, a single iron indicator (such as iron, ferritin, or hepcidin) only reflects a part of the body’s iron status. Therefore, a full assessment of the body’s iron status requires the combination of several iron indicators to provide a comprehensive understanding. Previous studies have primarily focused on the associations between iron deficiency and birth outcomes [6, 17, 34]. However, the effects of iron overload on fetal growth have received less attention. Encouraging further studies can provide a better understanding of the relationship between iron overload and fetal growth.

The objective of our study was to examine the relationship between maternal serum iron indicators (iron, sTFR, ferritin, hepcidin, and transferrin) during early pregnancy and neonatal anthropometric indicators (femur length, birth weight, body length, head circumference, and chest circumference) in a sample of 914 pregnant women. It was hypothesized that iron overload during early pregnancy could hinder fetal growth and be linked to lower neonatal anthropometric indicators.

## Methods

### Subjects

This study is a pilot analysis of a prospective cohort study conducted at Foshan Women and Children Hospital in Foshan City, Guangdong Province, China. The study is ongoing, and subjects were recruited for this pilot study from August 14, 2019, to January 31, 2021. Women who are 18 years of age or older and have been diagnosed as pregnant in the first trimester (up to 14 weeks’ gestation) by a medical professional and are planning to deliver their babies were recruited. Subjects with diabetes, serious illnesses that interfere with normal life such as cardiovascular disease, chronic kidney disease, and cancer, as well as mental disorders, were preliminarily excluded. A total of 987 subjects had their serum concentrations of iron-related indicators measured. Of these, 73 subjects were further excluded for the following reasons: (1) twins or multiple pregnancy (27 subjects); (2) with blood samples collected after 14 weeks of gestation (11 subjects); (3) missing core data (35 subjects). Finally, 914 subjects were included in this pilot study. All subjects provided written informed consent. The study was conducted in accordance with the Declaration of Helsinki and was approved by the Ethics Committee of Foshan Women and Children Hospital (FSFY-MEC-2019-025).

### Measurement of iron-related indicators

Participants in this study were personally interviewed and had a blood sample collected during their initial antenatal visit in the first trimester. Venous blood was collected by professional nurses. Serum samples were separated within less than 6 h and stored at -80℃ until analysis. The serum concentrations of iron-related indicators were measured using the enzyme-linked immunosorbent assay (ELISA) method. The ELISA Kit for serum iron (A039-1-1) was purchased from Jiancheng Biological Engineering Research Institute (Nanjing, China), while the ELISA kits for serum sTFR (CSB-E09100h), ferritin (CSB-E05187h), and hepcidin (CSB-E13062h) were purchased from Huamei Biological Engineering Co., LTD (Wuhan, China). The ELISA kit for serum transferrin was purchased from Elabscience Biotechnology Co., Ltd (Wuhan, China). The experimental procedure was conducted in strict accordance with the product instructions and verified by the methodology. The coefficient of variation (CV) values of the serum iron-related indicators ranged from 2.8 to 5.0% among the inter-board quality control samples and from 2.90 to 4.18% among 11 mixed serum quality control samples.

### Outcomes

The study outcomes included neonatal anthropometric indicators, such as birth weight, body length, head circumference, and chest circumference, as well as infant femur length. The length of the femur was measured shortly before delivery using B-ultrasound by specialist doctors. Neonatal anthropometric indicators were measured and recorded on the neonatal record sheet at delivery, and data were collected from the medical record. For the purposes of this study, LBW was defined as a birth weight of 2500 g or less, while macrosomia was defined as a birth weight exceeding 4000 g.

### Potential covariates

A structured questionnaire was used at baseline to collect general demographic and socioeconomic data, including history of gravidity and parity, maternal education (senior high school or below, junior college, bachelor’s or above), household income (≤ 5000, 5000–10,000, > 10,000 yuan per month), and use of iron or multivitamin supplements before pregnancy (yes or no). Maternal height was measured to the nearest 0.1 cm with the subjects wearing light clothing and no shoes. Maternal pre-pregnancy weight was self-reported, as were paternal height and average weight in the last three months. Mode of delivery and infant sex were obtained from medical records after the subjects delivered their babies.

### Statistical analysis

Continuous variables are presented as mean and SD or median and interquartile range as appropriate. Categorical variables are expressed as numbers and percentages. Serum concentrations of iron-related indicators were Z-normalized and divided into extreme 10th percentiles (< 10th, 10th-90th, and > 90th percentiles) as appropriate. Subjects with serum concentrations of iron-related indicators within the 10th-90th percentile were considered normal, while those below the 10th percentile were classified as extremely low and those above the 90th percentile were classified as extremely high.

Linear regression analyses were used to explore potential linear associations between serum iron-related indicators (per one standard deviation, SD increase) and neonatal anthropometric indicators. Cox regression analyses were used to explore associations between serum iron-related indicators (per one SD increase or extreme 10th percentiles) and LBW/macrosomia. The follow-up period was defined as the range of gestational weeks between the measurement of iron-related indicators and delivery. Analyses of covariance (ANCOVA) were used to compare the mean differences in femur length and other neonatal anthropometric indicators between groups of percentiles of serum iron-related indicators. Bonferroni correction was used for multiple comparisons in the ANCOVA analyses. Two different adjustment models were used in this study. Model 1 adjusted for age and gestational age (with femur length measurement weeks also adjusted for femur length). Model 2 further adjusted for mode of delivery, gravidity, parity, maternal height, maternal pre-pregnancy weight, paternal height, paternal weight, fetal sex, and measurement time of iron-related indicators (which were excluded for Cox regression analyses), as well as the use of iron or multivitamin supplements before pregnancy. The statistical analyses were conducted using SPSS 21.0 for Windows (SPSS, Inc., Chicago, USA) and R version 4.3.1. A significance level of 0.05 was used.

## Results

This prospective study included 914 Chinese mother-child dyads. The mean age of the mothers was 30.14 ± 4.39 years, and the mean gestational age was 39.03 ± 1.47 weeks (Table 1). The distribution of neonatal sex was 476 (52.1%) boys and 438 (47.9%) girls. Iron-related indicators’ serum concentrations were measured from blood samples collected in early pregnancy at a mean gestational age of 10.63 ± 1.56 weeks. The femur length was measured closest to delivery at a mean of 38.15 ± 1.91 weeks’ gestation.

**Table 1:** Characteristic of subjects

	*N* = 914
Age, years	30.14 ± 4.39
Maternal height, cm	158.4 ± 5.20
Maternal pre-pregnancy weight, kg	51.57 ± 7.51
Paternal height, cm	171.2 ± 5.40
Paternal weight, kg	67.95 ± 10.37
Gestational age, gestational weeks	39.03 ± 1.47
Measurement time of iron-related indicators, gestational weeks	10.63 ± 1.56
Measurement time of femur length, gestational weeks	38.15 ± 1.91
Gravidity, times	2.13 ± 1.21
Parity, times	1.50 ± 0.61
Maternal education	
Senior high school or below	362 (39.6)
Junior college	301 (32.9)
Bachelor or above	251 (27.5)
Household income, Yuan per month	
≤ 5000	353 (38.6)
5000–10,000	398 (43.5)
> 10,000	163 (17.8)
Neonatal s	
Boy	476 (52.1)
Girl	438 (47.9)
Delivery mode	
Natural labour, *n* (%)	498 (54.5)
Caesarean section, *n* (%)	416 (45.5)
Iron or multivitamin supplements (before pregnancy)	
Yes	46 (5.0)
No	868 (95.0)

To investigate the relationship between iron metabolism disorders and neonatal birth outcomes, we defined normal subjects as those with serum concentrations of iron-related indicators between the 10th and 90th percentile. Those with serum concentrations below the 10th percentile were classified as having extremely low levels, while those with serum concentrations above the 90th percentile were classified as having extremely high levels. Table 2 displays the distributions of serum concentrations of iron-related indicators at different percentiles (< 10th, 10th-90th, > 90th).

**Table 2:** Extreme 10th percentile distribution of serum concentrations of iron-related indicators

	Serum concentrations of iron-related indicators		
median (IQR)	*10th-90th percentile*	*< 10 th percentile*	*> 90th percentile*
	*N* = 701	*N* = 102	*N* = 90
Iron, mg/L	2.39 (2.05, 2.77)	1.39 (1.25, 1.54)	3.43 (3.29, 3.68)
	*N* = 733	*N* = 90	*N* = 91
sTFR, µg/L	2761 (1921, 3958)	603 (405, 795)	7211 (6567, 8499)
Ferritin, µg/L	90.3 (59.3, 131)	15.5 (8.62, 19.6)	259 (227, 286)
Hepcidin, µg/L	188 (124, 263)	42.3 (29.5, 54.0)	418 (387, 461)
Transferrin, ng/L	657 (446, 1016)	129 (103, 170)	2142 (1870, 2791)

sTFR: Soluble transferrin receptor

Linear regression analyses were used to investigate potential linear associations between exposure and birth outcomes, as shown in Table 3. After adjusting for potential covariates, an increase of one SD increase in serum iron concentration was associated with a 0.22 mm decrease in femur length. On the other hand, for transferrin, each one SD increase was associated with a 0.20 mm increase in femur length. No significant linear associations were observed between iron-related indicators and other anthropometric indicators, including birth weight, body length, head circumference, and chest circumference.

**Table 3:** Associations of serum concentrations of iron-related indicators with neonatal anthropometric indicators

	Per one standard deviation increase (SD)														
	Femur length, mm			Birth weight, g			Body length, cm			Head circumference, cm			Chest circumference, cm		
	*β*	*se*	*p*	*β*	*se*	*p*	*β*	*se*	*p*	*β*	*se*	*p*	*β*	*se*	*p*
Iron, mg/L															
Model 1	-0.21	0.078	**0.007**	-19.51	11.9	0.102	-0.10	0.074	0.165	-0.07	0.062	0.275	-0.11	0.066	0.099
Model 2	-0.22	0.077	**0.005**	-16.61	11.2	0.139	-0.08	0.072	0.256	-0.05	0.060	0.424	-0.10	0.064	0.128
sTFR, µg/L															
Model 1	0.13	0.076	0.085	-4.36	11.7	0.711	-0.03	0.072	0.707	-0.08	0.060	0.163	-0.01	0.064	0.921
Model 2	0.14	0.076	0.062	7.87	11.2	0.484	0.02	0.071	0.800	-0.01	0.059	0.925	0.06	0.064	0.333
Ferritin, µg/L															
Model 1	0.02	0.076	0.838	-17.00	11.8	0.149	-0.11	0.072	0.121	-0.09	0.060	0.144	-0.04	0.065	0.554
Model 2	0.04	0.076	0.651	-4.40	11.3	0.697	-0.06	0.070	0.379	-0.01	0.059	0.936	0.03	0.064	0.620
Hepcidin, µg/L															
Model 1	0.10	0.076	0.179	-10.00	11.7	0.393	-0.06	0.072	0.393	-0.09	0.060	0.146	-0.05	0.064	0.481
Model 2	0.11	0.077	0.141	3.86	11.3	0.733	-0.01	0.072	0.906	0.00	0.059	0.981	0.03	0.064	0.663
Transferrin, ng/L															
Model 1	0.16	0.076	**0.030**	1.76	11.7	0.881	0.04	0.072	0.547	-0.08	0.060	0.197	0.01	0.064	0.841
Model 2	0.20	0.076	**0.010**	16.90	11.2	0.132	0.11	0.071	0.130	0.01	0.059	0.883	0.09	0.063	0.160

Model 1: adjusted age and gestational age (for femur length, femur length measurement weeks were adjusted); Model 2: further adjusted for delivery mode, gravidity, parity, maternal height, maternal pre-pregnancy weight, paternal height, paternal weight, fetus’s sex, measurement time of iron-related indicators, use of iron or multivitamin supplements before pregnancy

**Fig. 1:**
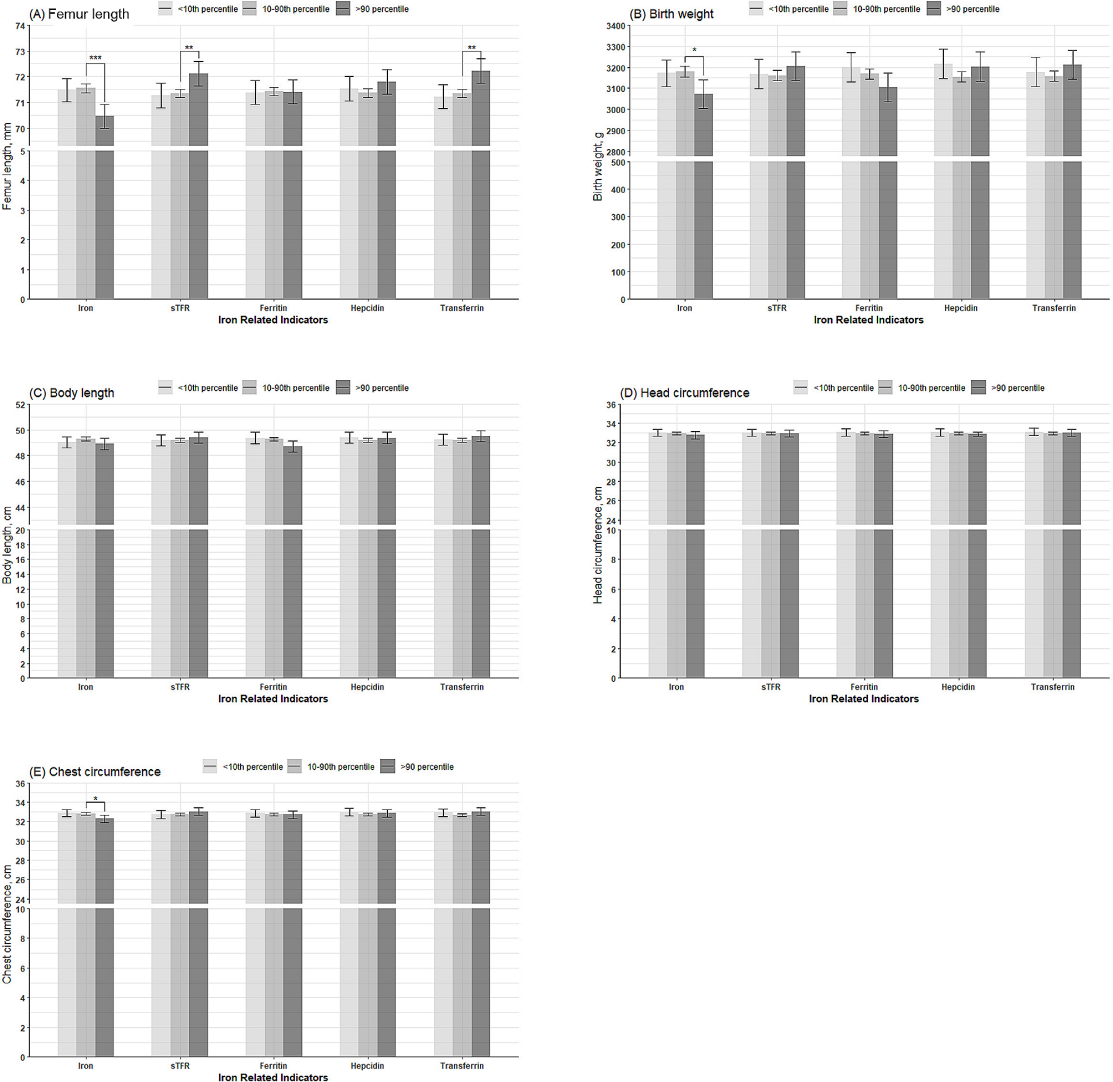
Associations of the extremely 10th percentile of serum concentrations of iron-related indicators with neonatal anthropometric indicators, including: **(A)** femur length, measured by B-mode ultrasound nearest delivery; **(B)** birth weight; **(C)** body length; **(D)** head circumference; and **(E)** chest circumference. Analyses of covariance were used to compare the differences between groups of < 10th, 10th-90th, and > 90th percentiles of serum concentrations of iron-related indicators, after adjusting for potential covariates. Data were presented as adjusted means. Error bars in the figure presented standard errors. Multiple comparisons among groups were adjusted using Bonferroni correction. *: *P* < 0.05; **: *P* < 0.01; ***: *P* < 0.001

ANCOVA analyses and Bonferroni corrections for multiple comparisons were used to compare results between different groups at the extreme 10th percentile of iron-related indicators. The results are presented in Fig. 1. Supplemental Tables 1–5 contain detailed data of the ANCOVA analyses. The study found that subjects with iron concentration levels above the 90th percentile had a 1.09 mm shorter femur length (1.52% of the mean in the normal group), a 105.8 g lower birth weight (3.33% of the mean in the normal group), and a 0.52 cm smaller chest circumference (1.58% of the mean in the normal group) compared to normal subjects (10th-90th percentiles). Compared to normal subjects, those with high concentrations (> 90th percentile) of sTFR and transferrin were associated with a 0.76 mm (0.99% of the mean in the normal group) and a 0.88 mm (1.14% of the mean in the normal group) greater femur length, respectively.

**Fig. 2:**
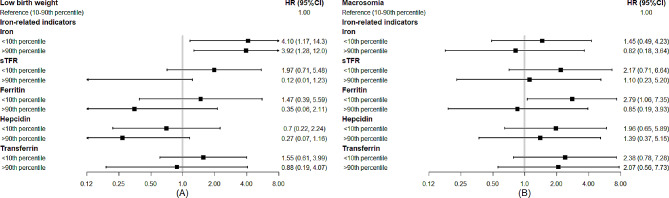
Associations of serum concentrations of iron-related indicators with the risk of low birth weight **(A)** and macrosomia **(B)**

Cox regression analyses were used to explore the associations between iron-related indicators and birth outcomes of LBW and macrosomia, as shown in Fig. 2. Further information can be found in Supplemental Tables 6–7. After adjusting for potential covariates, individuals with high serum iron concentration (> 90th percentile) were found to have a higher risk of LBW (HR: 3.92, 95% CI: 1.28, 12.0) compared to those with normal levels. Individuals with low iron and ferritin concentration (< 10th vs. 10th-90th percentile) were associated with a higher risk of LBW (HR: 4.10, 95% CI: 1.17, 14.3) and macrosomia (HR: 2.79, 95% CI: 1.06, 7.35), respectively. No significant associations were observed for other iron-related indicators (< 10th or > 90th percentile vs. 10th-90th percentile) with LBW or macrosomia. Our study did not find any significant associations between iron-related indicators (per one SD increase) and LBW or macrosomia.

## Discussion

This prospective cohort study of 914 Chinese mother-child dyads found that maternal iron overload in early pregnancy may have a negative impact on neonatal anthropometric indicators and birth outcomes. Elevated serum iron levels above the 90th percentile were linked to reduced neonatal anthropometric indicators, including femur length, birth weight, and chest circumference, as well as an increased risk of LBW. Compared to individuals with normal levels (10th-90th percentile) of sTFR and transferrin, those with high concentrations (> 90th percentile) showed a higher femur length. Conversely, individuals with low iron and ferritin concentrations (< 10th percentile) had a higher risk of LBW and macrosomia, respectively.

Our study found that individuals with iron overload status had a lower femur length. This negative association between iron overload and bone health has been supported by several other studies. In the MOCEH study of 337 women, it was found that higher maternal iron intake (top vs. middle tertile) was associated with a 0.41 cm lower femur length (*P* = 0.019) [28]. In South Korea, there was an observed correlation between elevated serum ferritin levels and decreased bone mineral density (BMD) as well as weakened femur neck strength, in two separate studies involving 693 women aged ≥ 45 years [27] and 4000 women aged 12 to 49 years [35]. Additionally, higher serum ferritin levels were associated with accelerated bone loss rates over a span of three years in 940 postmenopausal women and 789 middle-aged men [36]. Contrary to our findings, there were no statistically significant correlations found between the concentration of iron in amniotic fluid at 15.7 ± 1.1 weeks of gestation and femur length during the gestational periods of 16–20 and 32–36 weeks [26]. Several possible mechanisms may help to explain the negative association between iron overload and bone health. Iron overload inhibits bone formation and increases bone resorption by increasing oxidative stress and the expression of RANKL and IL-6 [37]. Iron overload can lead to endocrine dysfunction, such as hypothyroidism, higher HbA1c levels, and lower adrenocorticotropin levels, which can also affect bone health [38]. In addition, iron overload may shape the bone through iron-related proteins (e.g. transferrin receptor 2, hemojuvelin and bone morphogenetic protein) [39].

We observed that both excessively high (> 90th percentile, vs. 10th-90th percentile) or excessively low iron status (< 10th percentile, vs. 10th-90th percentile) were associated with a higher risk of LBW. Our finding was consistent with an earlier study of 1,218 pregnant women in Colombia, which found that serum ferritin depletion and gestational anemia in late pregnancy contributed to a lower LBW risk, and each 1 g/dL increase in hemoglobin was associated with a 36.8 g lower birth weight [32]. Several studies have suggested that a higher dietary intake of iron or iron supplementation, with or without folic acid, is associated with a higher birth weight or a lower risk of LBW [6, 13, 14]. A null association between iron supplementation and birth weight was also found in an RCT of 119 Danish women [40]. A study of 1660 women in rural Bangladesh found U-shaped associations between maternal hemoglobin levels and the risk of LBW [33]. It is possible that U-shaped associations also exist between maternal iron status and birth weight or LBW. More studies are needed to further examine our findings, and more attention should be paid to iron overload, which has been relatively understudied.

We discovered that elevated maternal serum iron levels (> 90th percentile vs. 10th-90th percentile) were associated with a smaller chest circumference. In contrast, in a double-blind randomized community trial of 4130 women in rural Nepal, iron supplementation with folic acid was positively associated with chest circumference [21]. No significant results were found for body length/height and head circumference in our study. Although higher ferritin levels (> 90th percentile vs. 10th-90th percentile) tended to be associated with shorter body length, the p-value is marginal (*p* = 0.055) rather than significant. Our findings were supported by several studies that found no significant associations between iron exposure and body length/height [25, 41] or head circumference [22, 23, 25]. However, other studies have found positive [7, 8, 19] or negative [29–31] associations between iron exposure and these two anthropometric indicators. Sources of heterogeneity in these studies may include different routes of iron exposure (including diet, iron supplementation, blood, and placenta), different study populations (in some studies, outcomes were measured in children rather than fetal or neonatal), and differences in baseline iron levels in the developed and developing countries studied, such as Sweden and Honduras [29].

In general, our findings suggested that maternal iron overload status may inhibit fetal growth. With the ability to modulate epigenetic factors and relevant pathways [42, 43], iron overload has been adversely associated with both early growth [44] and long-term health, such as metabolic diseases [45], aging [46], and immune diseases [43]. Some of the adverse influences (such as LBW and metabolic diseases) can be transmitted to future generations [47]. More attention should be paid to avoiding iron overload to maintain health. Our results, together with previous evidence, emphasize that more attention should be paid to the influence of maternal iron overload on fetal growth. Given the large body of evidence about the association between iron deficiency and its detrimental effects on fetal and postnatal growth and development, the influence of ID cannot be, then, ignored [3, 4]. More research is needed to explore appropriate iron ranges and iron supplementation strategies to reduce the adverse effects of maternal and infant iron deficiency, and to reduce the adverse effects of iron overload on fetal and infant growth. Our study suggests that maternal dietary iron excess may inhibit fetal growth; however, this is only a single-center study based on a Chinese population. Further research is warranted to establish a more robust body of evidence for this finding, as well as to gain a comprehensive understanding of the optimal range of essential nutrients in infant products (such as formula) [48] for diverse populations, considering variations in lifestyles, cultures, diets, and supplement usage. This necessitates conducting subsequent multicenter observational and interventional studies involving larger and more diverse populations.

### Strength and limitations

Our study has several strengths. First, considering the prospective design of this cohort study and the fact that serum concentrations of iron-related indicators were measured using blood samples collected in early pregnancy (≤ 14 weeks of gestation), the temporal sequence of events could be ensured, and possible causal inversions could be avoided. Second, several iron-related indicators (including iron, sTFR, ferritin, hepcidin, transferrin) were measured and analyzed, which may provide a more comprehensive understanding of the associations between maternal iron metabolism and infant growth, including several anthropometric indicators (e.g. femur length, birth weight, body length). Third, by comparing subjects with normal (10th-90th percentile) or extreme (> 90th or < 10th percentile) concentrations of iron-related indicators, our study was able to present the adverse association between iron metabolism imbalance and infant growth, and further emphasizes the importance of maintaining iron metabolism during pregnancy.

Several disadvantages in this study merit careful consideration. First, our study was based on a single obstetric hospital in Foshan City, China. Although this hospital is the largest obstetric center in this city and covers a large population, there may be a potential selection bias. Further studies based on multiple centers and more representative populations are needed. Second, the exposures (serum concentrations of iron metabolism indicators) were measured only once during early pregnancy. Therefore, we were not able to explore associations between exposures over time and neonatal birth outcomes. Two or three different assessments over the course of pregnancy would likely provide more comprehensive data, potentially useful in finding new strategies for appropriate iron supplementation throughout pregnancy. Further studies focusing on iron metabolism during the second and/or third trimesters and neonatal birth outcomes are needed to provide a more complete understanding in this area. Finally, there may still be confounding, although a number of potential covariates were controlled for in our study.

## Conclusion

In this prospective cohort study, we found that maternal iron overload in early pregnancy (≤ 14 weeks’ gestation) may adversely contribute to neonatal anthropometric indicators and adverse birth outcomes. Our study suggests that an imbalance in iron metabolism (both extremely high and low) may be associated with adverse infant growth, and emphasizes the importance of maintaining a balanced iron metabolism during pregnancy. Further high-quality studies are needed to confirm our study.

### Electronic supplementary material

Below is the link to the electronic supplementary material.

Supplementary Material 1

## Data Availability

The datasets used and/or analyzed during the current study are available from the corresponding author on reasonable request.

## Abbreviations

FGR: fetal growth restriction
ELISA: enzyme-linked immunosorbent assay
RCT: randomized controlled trial
LBW: low birth weight
SD: standard deviation
sTFR: serum transferrin receptor
CV: coefficient of variation
ANCOVA: analyses of covariance
