# Supplementary material for: Associations of maternal serum concentration of iron-related indicators with birth outcomes in Chinese: a pilot prospective cohort study
Source: Ital J Pediatr. 2024 Mar 5;50:39. doi: 10.1186/s13052-024-01621-0 (PMC10913255; doi:10.1186/s13052-024-01621-0)
Supplement: Supplementary file 1 — Supplementary Material 1 [file 13052_2024_1621_MOESM1_ESM.docx]

**Supplemental Table-1** Associations of extreme 10th percentile of serum concentrations of iron-related indicators with femur length.

| Femur length,mm | Serum concentrations of iron-related indicators | | |  |  |  |  |  |  |
| --- | --- | --- | --- | --- | --- | --- | --- | --- | --- |
|  | *10**th-90th percentile* | *<10 th percentile* | *>90th percentile* | *Diff 1^a^* | *Diff% 1^a^* | P-Diff 1*^a^* | Diff 2^b^ | *Diff% 2^b^* | P-Diff 2^b^ |
| Iron, mg/L |  |  |  |  |  |  |  |  |  |
| Model Ⅰ ^d^ | 72.54 ± 0.086 | 71.49 ± 0.227 | 70.50 ± 0.241 | -0.056 | -0.08 | 1.000 | -1.041 | -1.44 | **<0.001** |
| Model Ⅱ ^e^ | 71.55 ± 0.085 | 71.48 ± 0.224 | 70.46 ± 0.238 | -0.074 | -0.10 | 1.000 | -1.087 | -1.52 | **<0.001** |
| sTFR, μg/L | |  |  |  |  |  |  |  |  |
| Model Ⅰ ^d^ | 71.36 ± 0.084 | 71.28 ± 0.241 | 72.10 ± 0.240 | -0.072 | -0.10 | 1.000 | 0.741 | 1.04 | **0.011** |
| Model Ⅱ ^e^ | 71.36 ± 0.083 | 71.27 ± 0.239 | 72.12 ± 0.238 | -0.087 | -0.12 | 1.000 | 0.759 | 0.99 | **0.008** |
| Ferritin, μg/L |  |  |  |  |  |  |  |  |  |
| Model Ⅰ ^d^ | 71.44 ± 0.085 | 71.35 ± 0.242 | 71.38 ± 0.241 | -0.083 | -0.12 | 1.000 | -0.052 | -0.07 | 1.000 |
| Model Ⅱ ^e^ | 71.43 ± 0.083 | 71.38 ± 0.240 | 71.41 ± 0.239 | -0.047 | -0.07 | 1.000 | -0.02 | -0.15 | 1.000 |
| Hepcidin, μg/L |  |  |  |  |  |  |  |  |  |
| Model Ⅰ ^d^ | 71.37 ± 0.085 | 71.49 ± 0.240 | 71.77 ± 0.242 | 0.116 | 0.16 | 1.000 | 0.393 | 0.55 | 0.374 |
| Model Ⅱ ^e^ | 71.36 ± 0.083 | 71.53 ± 0.240 | 71.79 ± 0.240 | 0.165 | 0.23 | 1.000 | 0.426 | 0.51 | 0.285 |
| Transferrin, ng/L | |  |  |  |  |  |  |  |  |
| Model Ⅰ ^d^ | 71.36 ± 0.084 | 71.18 ± 0.239 | 72.16 ± 0.239 | -0.178 | -0.25 | 1.000 | 0.804 | 1.13 | **0.005** |
| Model Ⅱ ^e^ | 71.35 ± 0.083 | 71.22 ± 0.239 | 72.22 ± 0.237 | -0.126 | -0.18 | 1.000 | 0.876 | 1.14 | **0.002** |

^a^: *Diff 1*: difference between groups of <10th and 10-90th percentile，*Diff% 1*=100×Diff 1/Mean(10-90th percentile), *P-Diff 1* represented p value for the difference; ^b^: *Diff 2*: difference between groups of >90 th and 10-90th percentile，*Diff% 2*=100×Diff 2/Mean(10-90th percentile), *P-Diff 2* represented p value for the difference; ACONVA analyses: Model 1: adjusted age and femur length measurement weeks; Model 2: further adjusted for delivery mode，gravidity, parity, maternal height, maternal pre-pregnancy weight, paternal height，paternal weight, fetus’s sex, measurement time of iron-related indicators，use of iron or multivitamin supplements before pregnancy. Multiple comparison among groups were adjusted using Bonferroni methods.

**Supplemental Table-2** Associations of extreme 10th percentile of serum concentrations of iron-related indicators with birth weight.

| Birth weight, g | Serum concentrations of iron-related indicators | | |  |  |  |  |  |  |
| --- | --- | --- | --- | --- | --- | --- | --- | --- | --- |
|  | *10th-90th percentile* | *<10 th percentile* | *>90th percentile* | *Diff 1^a^* | *Diff% 1^a^* | P-Diff 1*^a^* | Diff 2^b^ | *Diff% 2^b^* | P-Diff 2^b^ |
| Iron, mg/L |  |  |  |  |  |  |  |  |  |
| Model Ⅰ ^d^ | 3177 ± 13.25 | 3182 ± 34.83 | 3071 ± 36.99 | 4.575 | 0.14 | 1.000 | -106 | -3.34 | **0.021** |
| Model Ⅱ ^e^ | 3179 ± 12.41 | 3171 ± 32.83 | 3072 ± 34.80 | -7.700 | -0.24 | 1.000 | -105.8 | -3.33 | **0.013** |
| sTFR, μg/L | |  |  |  |  |  |  |  |  |
| Model Ⅰ ^d^ | 3160 ± 13.13 | 3192 ± 37.49 | 3178 ± 37.25 | 32.62 | 1.03 | 1.000 | 18.03 | 0.57 | 1.000 |
| Model Ⅱ ^e^ | 3160 ± 12.27 | 3167 ± 35.40 | 3205 ± 35.14 | 7.141 | 0.23 | 1.000 | 44.9 | 1.42 | 0.685 |
| Ferritin, μg/L |  |  |  |  |  |  |  |  |  |
| Model Ⅰ ^d^ | 3168 ± 13.09 | 3219 ± 37.35 | 3089 ± 37.19 | 51.31 | 1.62 | 0.586 | -79.04 | -2.49 | 0.136 |
| Model Ⅱ ^e^ | 3168 ± 12.26 | 3200 ± 35.33 | 3105 ± 35.10 | 32.47 | 1.02 | 1.000 | -63.07 | -1.99 | 0.271 |
| Hepcidin, μg/L |  |  |  |  |  |  |  |  |  |
| Model Ⅰ ^d^ | 3154 ± 13.10 | 3237 ± 37.18 | 3177 ± 37.38 | 83.05 | 2.63 | 0.106 | 22.49 | 0.71 | 1.000 |
| Model Ⅱ ^e^ | 3154 ± 12.27 | 3216 ± 35.31 | 3202 ± 35.40 | 62.19 | 1.97 | 0.291 | 48.84 | 1.55 | 0.580 |
| Transferrin, ng/L | |  |  |  |  |  |  |  |  |
| Model Ⅰ ^d^ | 3160 ± 13.13 | 3190 ± 37.27 | 3175 ± 37.25 | 29.94 | 0.95 | 1.000 | 15.14 | 0.48 | 1.000 |
| Model Ⅱ ^e^ | 3157 ± 12.28 | 3177 ± 35.35 | 3211 ± 35.11 | 20.01 | 0.63 | 1.000 | 53.55 | 1.70 | 0.451 |

^a^: *Diff 1*: difference between groups of <10th and 10-90th percentile，*Diff% 1*=100×Diff 1/Mean(10-90th percentile), *P-Diff 1* represented p value for the difference; ^b^: *Diff 2*: difference between groups of >90 th and 10-90th percentile，*Diff% 2*=100×Diff 2/Mean(10-90th percentile), *P-Diff 2* represented p value for the difference; ACONVA analyses: Model 1: adjusted age and gestational age; Model 2: further adjusted for delivery mode，gravidity, parity, maternal height, maternal pre-pregnancy weight, paternal height，paternal weight, fetus’s sex, measurement time of iron-related indicators，use of iron or multivitamin supplements before pregnancy. Multiple comparison among groups were adjusted using Bonferroni methods.

**Supplemental Table-3** Associations of extreme 10th percentile of serum concentrations of iron-related indicators with body length.

| Body length, cm | Serum concentrations of iron-related indicators | | |  |  |  |  |  |  |
| --- | --- | --- | --- | --- | --- | --- | --- | --- | --- |
|  | *<10 th percentile* | *10th-90th percentile* | *>90th percentile* | *Diff 1^a^* | *Diff% 1^a^* | P-Diff 1*^a^* | Diff 2^b^ | *Diff% 2^b^* | P-Diff 2^b^ |
| Iron, mg/L |  |  |  |  |  |  |  |  |  |
| Model Ⅰ ^d^ | 49.28 ± 0.08 | 49.08 ± 0.22 | 48.86 ± 0.23 | -0.204 | -0.41 | 1.000 | -0.424 | -0.86 | 0.248 |
| Model Ⅱ ^e^ | 49.29 ± 0.08 | 49.01 ± 0.21 | 48.89 ± 0.22 | -0.282 | -0.57 | 0.633 | -0.4 | -0.81 | 0.276 |
| sTFR, μg/L | |  |  |  |  |  |  |  |  |
| Model Ⅰ ^d^ | 49.20 ± 0.08 | 49.25 ± 0.23 | 49.31 ± 0.23 | 0.055 | 0.11 | 1.000 | 0.109 | 0.22 | 1.000 |
| Model Ⅱ ^e^ | 49.20 ± 0.08 | 49.17 ± 0.22 | 49.40 ± 0.22 | -0.032 | -0.07 | 1.000 | 0.206 | 0.42 | 1.000 |
| Ferritin, μg/L |  |  |  |  |  |  |  |  |  |
| Model Ⅰ ^d^ | 49.26 ± 0.08 | 49.42 ± 0.23 | 48.65 ± 0.23 | 0.158 | 0.32 | 1.000 | -0.613 | -1.24 | **0.034** |
| Model Ⅱ ^e^ | 49.26 ± 0.08 | 49.36 ± 0.22 | 48.70 ± 0.22 | 0.104 | 0.21 | 1.000 | -0.556 | -1.13 | 0.055 |
| Hepcidin, μg/L |  |  |  |  |  |  |  |  |  |
| Model Ⅰ ^d^ | 49.18 ± 0.08 | 49.43 ± 0.23 | 49.25 ± 0.23 | 0.245 | 0.50 | 0.931 | 0.068 | 0.14 | 1.000 |
| Model Ⅱ ^e^ | 49.18 ± 0.08 | 49.38 ± 0.22 | 49.36 ± 0.22 | 0.204 | 0.41 | 1.000 | 0.181 | 0.37 | 1.000 |
| Transferrin, ng/L | |  |  |  |  |  |  |  |  |
| Model Ⅰ ^d^ | 49.20 ± 0.08 | 49.23 ± 0.23 | 49.35 ± 0.23 | 0.034 | 0.07 | 1.000 | 0.155 | 0.32 | 1.000 |
| Model Ⅱ ^e^ | 49.18 ± 0.08 | 49.22 ± 0.22 | 49.49 ± 0.22 | 0.037 | 0.08 | 1.000 | 0.314 | 0.64 | 0.546 |

^a^: *Diff 1*: difference between groups of <10th and 10-90th percentile，*Diff% 1*=100×Diff 1/Mean(10-90th percentile), *P-Diff 1* represented p value for the difference; ^b^: *Diff 2*: difference between groups of >90 th and 10-90th percentile，*Diff% 2*=100×Diff 2/Mean(10-90th percentile), *P-Diff 2* represented p value for the difference; ACONVA analyses: Model 1: adjusted age and gestational age; Model 2: further adjusted for delivery mode，gravidity, parity, maternal height, maternal pre-pregnancy weight, paternal height，paternal weight, fetus’s sex, measurement time of iron-related indicators，use of iron or multivitamin supplements before pregnancy. Multiple comparison among groups were adjusted using Bonferroni methods.

**Supplemental Table-4** Associations of extreme 10th percentile of serum concentrations of iron-related indicators with head circumference.

| Head circumference, cm | Serum concentrations of iron-related indicators | | |  |  |  |  |  |  |
| --- | --- | --- | --- | --- | --- | --- | --- | --- | --- |
|  | *<10 th percentile* | *10th-90th percentile* | *>90th percentile* | *Diff 1^a^* | *Diff% 1^a^* | P-Diff 1*^a^* | Diff 2^b^ | *Diff% 2^b^* | P-Diff 2^b^ |
| Iron, mg/L |  |  |  |  |  |  |  |  |  |
| Model Ⅰ ^d^ | 32.96 ± 0.07 | 33.11 ± 0.18 | 32.78 ± 0.19 | 0.148 | 0.45 | 1.000 | -0.181 | -0.55 | 1.000 |
| Model Ⅱ ^e^ | 32.96 ± 0.07 | 33.01 ± 0.18 | 32.77 ± 0.19 | 0.095 | 0.29 | 1.000 | -0.19 | -0.58 | 1.000 |
| sTFR, μg/L | |  |  |  |  |  |  |  |  |
| Model Ⅰ ^d^ | 32.94 ± 0.07 | 33.22 ± 0.19 | 32.80 ± 0.19 | 0.272 | 0.83 | 0.540 | -0.143 | -0.43 | 1.000 |
| Model Ⅱ ^e^ | 32.95 ± 0.06 | 33.03 ± 0.19 | 32.96 ± 0.19 | 0.086 | 0.26 | 1.000 | 0.016 | 0.05 | 1.000 |
| Ferritin, μg/L |  |  |  |  |  |  |  |  |  |
| Model Ⅰ ^d^ | 32.95 ± 0.07 | 33.23 ± 0.19 | 32.76 ± 0.19 | 0.289 | 0.88 | 0.462 | -0.185 | -0.56 | 1.000 |
| Model Ⅱ ^e^ | 32.95 ± 0.06 | 33.06 ± 0.19 | 32.88 ± 0.19 | 0.106 | 0.32 | 1.000 | -0.078 | -0.24 | 1.000 |
| Hepcidin, μg/L |  |  |  |  |  |  |  |  |  |
| Model Ⅰ ^d^ | 32.95 ± 0.07 | 33.23 ± 0.19 | 32.76 ± 0.19 | 0.289 | 0.88 | 0.462 | -0.185 | -0.56 | 1.000 |
| Model Ⅱ ^e^ | 32.95 ± 0.06 | 33.06 ± 0.19 | 32.88 ± 0.19 | 0.106 | 0.32 | 1.000 | -0.078 | -0.24 | 1.000 |
| Transferrin, ng/L | |  |  |  |  |  |  |  |  |
| Model Ⅰ ^d^ | 32.93 ± 0.07 | 33.30 ± 0.19 | 32.83 ± 0.19 | 0.371 | 1.13 | 0.198 | -0.093 | -0.28 | 1.000 |
| Model Ⅱ ^e^ | 32.93 ± 0.07 | 33.11 ± 0.19 | 33.02 ± 0.18 | 0.198 | 0.19 | 1.000 | 0.088 | 0.27 | 1.000 |

^a^: *Diff 1*: difference between groups of <10th and 10-90th percentile，*Diff% 1*=100×Diff 1/Mean(10-90th percentile), *P-Diff 1* represented p value for the difference; ^b^: *Diff 2*: difference between groups of >90 th and 10-90th percentile，*Diff% 2*=100×Diff 2/Mean(10-90th percentile), *P-Diff 2* represented p value for the difference; ACONVA analyses: Model 1: adjusted age and gestational age; Model 2: further adjusted for delivery mode，gravidity, parity, maternal height, maternal pre-pregnancy weight, paternal height，paternal weight, fetus’s sex, measurement time of iron-related indicators，use of iron or multivitamin supplements before pregnancy. Multiple comparison among groups were adjusted using Bonferroni methods.

**Supplemental Table-5** Associations of extreme 10th percentile of serum concentrations of iron-related indicators with chest circumference.

| Chest circumference, cm | Serum concentrations of iron-related indicators | | |  |  |  |  |  |  |
| --- | --- | --- | --- | --- | --- | --- | --- | --- | --- |
|  | *<10 th percentile* | *10th-90th percentile* | *>90th percentile* | *Diff 1^a^* | *Diff% 1^a^* | P-Diff 1*^a^* | Diff 2^b^ | *Diff% 2^b^* | P-Diff 2^b^ |
| Iron, mg/L |  |  |  |  |  |  |  |  |  |
| Model Ⅰ ^d^ | 32.79 ± 0.07 | 32.90 ± 0.19 | 32.27 ± 0.20 | 0.113 | 0.34 | 1.000 | -0.52 | -1.59 | **0.050** |
| Model Ⅱ ^e^ | 32.79 ± 0.07 | 32.88 ± 0.19 | 32.28 ± 0.20 | 0.09 | 0.27 | 1.000 | -0.517 | -1.58 | **0.045** |
| sTFR, μg/L | |  |  |  |  |  |  |  |  |
| Model Ⅰ ^d^ | 32.71 ± 0.07 | 32.90 ± 0.21 | 32.89 ± 0.20 | 0.186 | 0.57 | 1.000 | 0.179 | 0.55 | 1.000 |
| Model Ⅱ ^e^ | 32.71 ± 0.07 | 32.73 ± 0.20 | 33.05 ± 0.20 | 0.015 | 0.05 | 1.000 | 0.335 | 1.02 | 0.336 |
| Ferritin, μg/L |  |  |  |  |  |  |  |  |  |
| Model Ⅰ ^d^ | 32.73 ± 0.07 | 32.98 ± 0.21 | 32.61 ± 0.20 | 0.246 | 0.75 | 0.771 | -0.119 | -0.36 | 1.000 |
| Model Ⅱ ^e^ | 32.74 ± 0.07 | 32.85 ± 0.20 | 32.72 ± 0.20 | 0.11 | 0.34 | 1.000 | -0.014 | -0.04 | 1.000 |
| Hepcidin, μg/L |  |  |  |  |  |  |  |  |  |
| Model Ⅰ ^d^ | 32.71 ± 0.07 | 33.11 ± 0.20 | 32.70 ± 0.20 | 0.409 | 1.25 | 0.174 | -0.003 | -0.01 | 1.000 |
| Model Ⅱ ^e^ | 32.71 ± 0.07 | 32.95 ± 0.20 | 32.84 ± 0.20 | 0.238 | 0.73 | 0.790 | 0.129 | 0.39 | 1.000 |
| Transferrin, ng/L | |  |  |  |  |  |  |  |  |
| Model Ⅰ ^d^ | 32.70 ± 0.07 | 33.03 ± 0.20 | 32.83 ± 0.20 | 0.336 | 1.03 | 0.361 | 0.136 | 0.42 | 1.000 |
| Model Ⅱ ^e^ | 32.69 ± 0.07 | 32.92 ± 0.20 | 33.02 ± 0.20 | 0.227 | 0.69 | 0.859 | 0.331 | 1.01 | 0.352 |

^a^: *Diff 1*: difference between groups of <10th and 10-90th percentile，*Diff% 1*=100×Diff 1/Mean(10-90th percentile), *P-Diff 1* represented p value for the difference; ^b^: *Diff 2*: difference between groups of >90 th and 10-90th percentile，*Diff% 2*=100×Diff 2/Mean(10-90th percentile), *P-Diff 2* represented p value for the difference; ACONVA analyses: Model 1: adjusted age and gestational age; Model 2: further adjusted for delivery mode，gravidity, parity, maternal height, maternal pre-pregnancy weight, paternal height，paternal weight, fetus’s sex, measurement time of iron-related indicators，use of iron or multivitamin supplements before pregnancy. Multiple comparison among groups were adjusted using Bonferroni methods.

**Supplemental Table-6** Associations of serum concentrations of iron-related indicators with risk of low birth weight.

|  | Risk of low birth weight | | | | | | | | | |
| --- | --- | --- | --- | --- | --- | --- | --- | --- | --- | --- |
|  | *10th-90th percentile* | *<10th percentile* | | | *>90th percentile* | | | Per one SD increase | | |
|  | *Reference* | *HR* | *95%CI* | *p* | *HR* | *95%CI* | *p* | *HR* | *95%CI* | *p* |
| Iron, mg/L |  |  |  |  |  |  |  |  |  |  |
| Model 1 | 1 | 1.36 | (0.44, 4.21) | 0.597 | **3.12** | **(1.24, 7.85)** | **0.016** | 1.15 | (0.80, 1.65) | 0.452 |
| Model 2 | 1 | **4.10** | **(1.17, 14.3)** | **0.027** | **3.92** | **(1.28, 12.0)** | **0.017** | 0.94 | (0.62, 1.44) | 0.788 |
| sTFR, μg/L | |  |  |  |  |  |  |  |  |  |
| Model 1 | 1 | 0.90 | (0.35, 2.36) | 0.837 | 0.17 | (0.02, 1.28) | 0.085 | 0.85 | (0.62, 1.18) | 0.334 |
| Model 2 | 1 | 1.97 | (0.71, 5.48) | 0.196 | 0.12 | (0.01, 1.23) | 0.074 | 0.73 | (0.48, 1.11) | 0.145 |
| Ferritin, μg/L |  |  |  |  |  |  |  |  |  |  |
| Model 1 | 1 | 0.51 | (0.14, 1.82) | 0.300 | 0.37 | (0.08, 1.62) | 0.186 | 0.91 | (0.65, 1.30) | 0.631 |
| Model 2 | 1 | 1.47 | (0.39, 5.59) | 0.572 | 0.35 | (0.06, 2.11) | 0.251 | 0.78 | (0.51, 1.20) | 0.265 |
| Hepcidin, μg/L |  |  |  |  |  |  |  |  |  |  |
| Model 1 | 1 | 0.55 | (0.18, 1.63) | 0.279 | 0.31 | (0.08, 1.26) | 0.101 | 0.86 | (0.63, 1.18) | 0.352 |
| Model 2 | 1 | 0.70 | (0.22, 2.24) | 0.543 | 0.27 | (0.07, 1.16) | 0.079 | 0.76 | (0.52, 1.11) | 0.152 |
| Transferrin, ng/L | |  |  |  |  |  |  |  |  |  |
| Model 1 | 1 | 1.10 | (0.45, 2.66) | 0.837 | 0.70 | (0.16, 3.02) | 0.633 | 0.78 | (0.52, 1.16) | 0.213 |
| Model 2 | 1 | 1.55 | (0.61, 3.99) | 0.359 | 0.88 | (0.19, 4.07) | 0.870 | 0.63 | (0.37, 1.07) | 0.085 |

Cox regression analyses; Model 1: adjusted age and gestational age (for femur length, femur length measurement weeks were adjusted); Model 2: further adjusted for delivery mode，gravidity, parity, maternal height, maternal pre-pregnancy weight, paternal height, paternal weight, fetus’s sex, use of iron or multivitamin supplements before pregnancy.

**Supplemental Table-7** Associations of serum concentrations of iron-related indicators with risk of macrosomia.

|  | Risk of macrosomia | | | | | | | | | |
| --- | --- | --- | --- | --- | --- | --- | --- | --- | --- | --- |
|  | *10th-90th percentile* | *<10th percentile* | | | *>90th percentile* | | | Per one SD increase | | |
|  | *Reference* | *HR* | *95%CI* | *p* | *HR* | *95%CI* | *p* | *HR* | *95%CI* | *p* |
| Iron, mg/L |  |  |  |  |  |  |  |  |  |  |
| Model 1 | 1.00 | 1.35 | (0.49, 3.74) | 0.564 | 0.71 | (0.16, 3.05) | 0.641 | 0.74 | (0.50, 1.11) | 0.142 |
| Model 2 | 1.00 | 1.45 | (0.49, 4.23) | 0.502 | 0.82 | (0.18, 3.64) | 0.792 | 0.76 | (0.50, 1.16) | 0.203 |
| sTFR, μg/L | |  |  |  |  |  |  |  |  |  |
| Model 1 | 1.00 | 2.23 | (0.78, 6.33) | 0.134 | 0.79 | (0.18, 3.48) | 0.755 | 0.88 | (0.58, 1.34) | 0.561 |
| Model 2 | 1.00 | 2.17 | (0.71, 6.64) | 0.176 | 1.10 | (0.23, 5.20) | 0.902 | 0.99 | (0.64, 1.51) | 0.945 |
| Ferritin, μg/L |  |  |  |  |  |  |  |  |  |  |
| Model 1 | 1.00 | **2.88** | **(1.15, 7.19)** | **0.024** | 0.93 | (0.21, 4.04) | 0.919 | 0.89 | (0.59, 1.33) | 0.557 |
| Model 2 | 1.00 | **2.79** | **(1.06, 7.35)** | **0.038** | 0.85 | (0.19, 3.93) | 0.839 | 0.96 | (0.65, 1.41) | 0.836 |
| Hepcidin, μg/L |  |  |  |  |  |  |  |  |  |  |
| Model 1 | 1.00 | 2.25 | (0.80, 6.35) | 0.127 | 1.32 | (0.38, 4.60) | 0.666 | 0.90 | (0.61, 1.34) | 0.602 |
| Model 2 | 1.00 | 1.96 | (0.65, 5.89) | 0.231 | 1.39 | (0.37, 5.15) | 0.627 | 0.99 | (0.65, 1.50) | 0.958 |
| Transferrin, ng/L | |  |  |  |  |  |  |  |  |  |
| Model 1 | 1.00 | 1.35 | (0.49, 3.74) | 0.564 | 0.71 | (0.16, 3.05) | 0.641 | 0.88 | (0.55, 1.40) | 0.585 |
| Model 2 | 1.00 | 2.38 | (0.78, 7.28) | 0.130 | 2.07 | (0.56, 7.73) | 0.278 | 0.96 | (0.60, 1.53) | 0.851 |

Cox regression analyses; Model 1: adjusted age and gestational age (for femur length, femur length measurement weeks were adjusted); Model 2: further adjusted for delivery mode, gravidity, parity, maternal height, maternal pre-pregnancy weight, paternal height，paternal weight, fetus’s sex, use of iron or multivitamin supplements before pregnancy.
